# Removal of Complete Spontaneous Migration of an Intrauterine Contraceptive Device to the Bladder by Cystoscopy

**DOI:** 10.1155/2024/6934178

**Published:** 2024-05-13

**Authors:** Akbar Novan Dwi Saputra, Muhammad Nurhadi Rahman, Anis Widyasari

**Affiliations:** Urogynecology and Reconstructive Surgery Division, Department of Obstetrics and Gynecology, Faculty of Medicine, Public Health and Nursing, Universitas Gadjah Mada, Yogyakarta, Indonesia

## Abstract

Intrauterine contraceptive devices (IUCD) are widely used as a reversible method of contraception. Although uterine perforation caused by an IUCD is rare, in some cases, the device may migrate to the pelvic/abdominal cavity or nearby structures. When the IUCD migrate to the bladder, it can lead to various symptoms. These include pain or discomfort in the lower abdomen, difficulties or pain during urination, frequent urinary tract infections, and the development of bladder stones. This article presents a case report of a 24-year-old woman with an intrauterine contraceptive device (IUCD) that had migrated completely into the bladder. She had an IUCD inserted by a midwife four years earlier and became pregnant a year later, unaware of the IUCD's presence. She later presented with lower abdominal pain, hematuria, and dysuria three months before being admitted to our hospital. Imaging confirmed the intravesical location of the IUCD. She underwent successful cystoscopy treatment to remove the migrated IUCD. Prompt diagnosis and appropriate management are crucial in preventing complications and improving patient outcomes. Clinicians should be aware of this potential complication and consider it when patients present with symptoms or complications after IUCD insertion. Regular monitoring and timely intervention can help detect and address IUCD migration effectively.

## 1. Introduction

In various regions globally, intrauterine contraceptive devices (IUCD) are widely employed as a commonly chosen and reversible method of contraception. In Eastern and South-Eastern Asia, the IUCD is the predominant choice for contraception, being the most widely utilized method. Approximately 18.6% of women in this region depend on the IUCD as their preferred contraceptive option [1]. IUCD is considered to be the most cost-effective contraceptive method in Indonesia, compared with other methods. The cost-effectiveness of the IUCD stems from its longevity, as a single device can provide protection for several years. This eliminates the need for frequent purchases of contraceptives, reducing overall expenses [2].

The IUCD provides long-term protection against unintended pregnancies and requires minimal maintenance, making it an attractive choice for many women. Its affordability, in addition to its effectiveness, has contributed to its popularity. Typically, their insertion is uncomplicated, with uterine perforation being a seldom-encountered issue. However, in rare cases, the IUCD may migrate to the pelvic/abdominal cavity or nearby structures following perforation [3]. The risk of uterine perforation caused by an IUCD is rare, occurring 1–3 times per 1000 insertions [4]. When an IUCD becomes displaced or migrates, it can lead to severe complications such as bowel perforation, hydronephrosis, and even renal failure. The occurrence of an IUCD translocating into the bladder is an uncommon event that may be linked to the lower urinary tract symptoms, formation of vesicouterine fistula, development of bladder stones, or bladder perforation [5]. Endoscopic treatments and open surgical removal are two therapeutic options for an intrauterine device in the bladder [6]. This article presents the case of a patient who underwent successful cystoscopy treatment for recurrent urinary tract infection caused by intrauterine contraceptive device migration.

## 2. Case Presentation

A 24-year-old female, Para 2002, visited the Gynecology Department at our hospital with complaints of lower abdominal pain, hematuria (blood in urine), and persistent pain during urination for the past three months. She mentioned that an intrauterine contraceptive device (IUCD) had been implanted by a midwife on January 12, 2019, and she had not experienced any issues or received any follow-up care since then. A year later, she became pregnant and delivered a baby vaginally. During her pregnancy, she primarily received antenatal care from a midwife and consulted with an obstetrician only twice. It is possible that during the ultrasound examination, the physician focused solely on intrauterine conditions and did not fully explore the pelvic cavity, leading to the missed diagnosis of an ectopic IUCD. As a result, she was unaware of the IUCD's presence at that time, assuming it had been expelled. She has no history of curettage, cesarean section, or other major gynecological surgeries.

**Figure 1:**
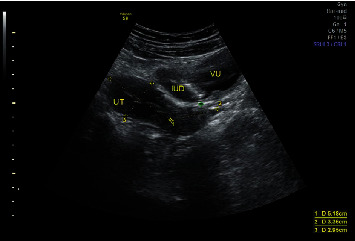
The abdominal ultrasound findings showed that all parts of the IUCD were located intravesically. UT: uterus; IUD: intrauterine contraceptive device; VU: vesica urinaria.

The pelvic examination showed normal results, but the urinary sediment indicated a urinary tract infection. Initially, she was diagnosed with cystitis and prescribed antibiotics by a general practitioner. However, her symptoms did not improve, leading to her being referred back to our department. During an ultrasound examination on February 2, 2023, no signs of an IUCD were found in the uterine cavity, but an IUCD was detected in the bladder (Figure 1). This indicated that the IUCD had perforated the uterus and migrated into the bladder.

**Figure 2:**
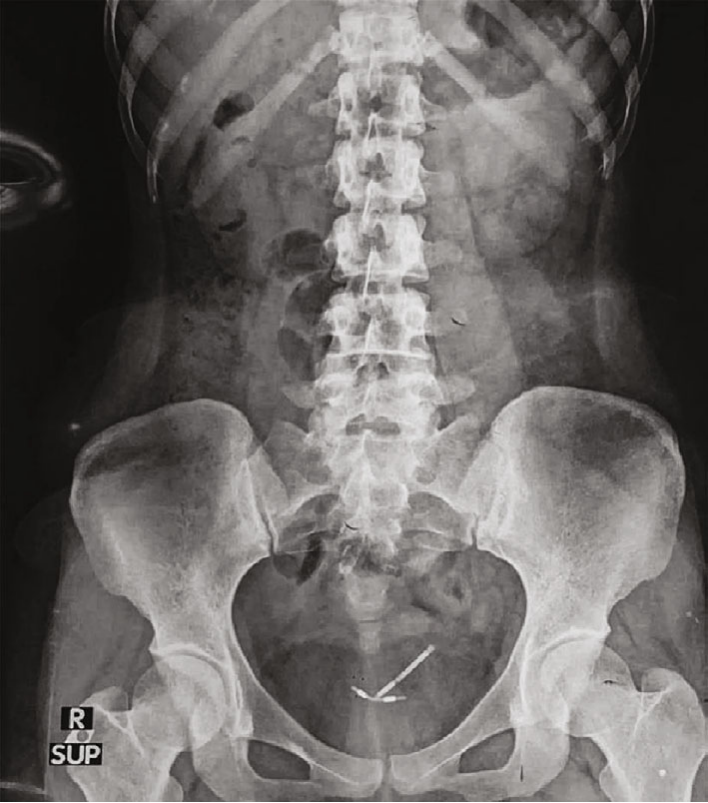
The abdominal plain X-ray revealed that the IUCD was visualized within the pelvic cavity (white arrow).

**Figure 3:**
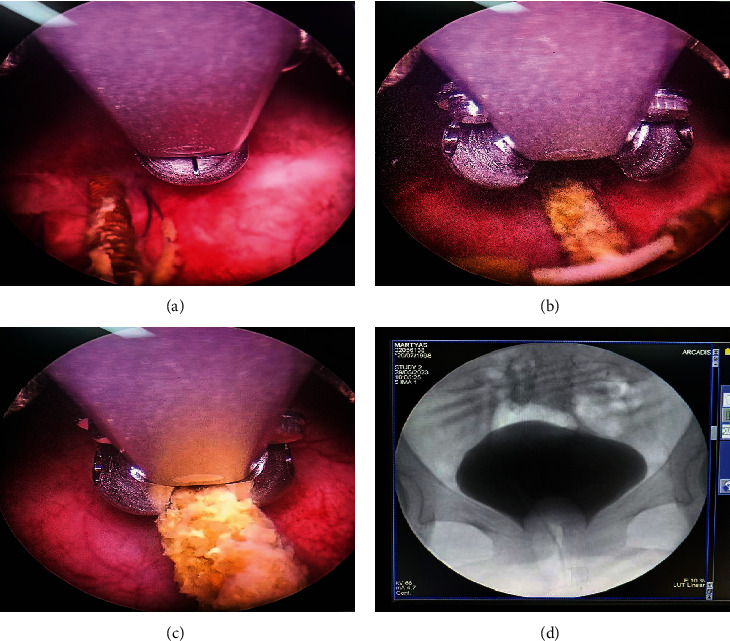
(a) The distal tip portion of IUCD (white arrow) embedded in the bladder posterior wall. (b) The IUCD with two short arms is attached to the posterior bladder wall. (c) The IUCD in the bladder being grasped with biopsy forceps. (d) No contrast extravasation observed on cystography postremoval.

**Figure 4:**
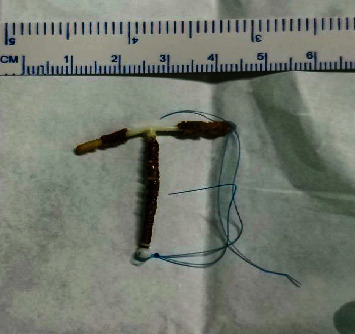
The IUCD after its removal.

The patient's laboratory tests showed normal results, but the urinalysis detected a significant presence of red blood cells and white blood cells. A plain abdominal X-ray revealed the presence of a T-shaped IUCD in the pelvic area (Figure 2). The cystoscopy procedure was conducted under regional anesthesia at our hospital. We utilized a 19 French cystoscope equipped with a 30° lens. During the cystoscopy, we observed that the intrauterine contraceptive device (IUCD) was located within the bladder. The distal tip of the IUCD was found to be embedded in the posterior wall of the bladder (Figure 3(a)), while the two short arms of the IUCD were adhered to the mucosal layer of the posterior bladder wall (Figure 3(b)). Encrustations were observed, but no calculi were detected in the bladder cavity. We successfully removed the IUCD using forceps guided by the cystoscope (Figures 3(c) and 4). Following the removal, we conducted a postremoval cystoscopy to reevaluate the bladder and ensure that there were no stones, foreign objects, or bleeding points present. Cystography showed no evidence of a fistula or other major defects on the bladder wall (Figure 3(d)). Subsequently, we emptied the bladder, and the surgical procedure was concluded. Following the operation, a Foley catheter was inserted, and the patient's recovery was without complications. The patient was discharged in good condition the day after the procedure, following catheter removal, without reporting any complications. During a follow-up visit, the patient showed no symptoms or significant clinical findings. We also offer patients hormonal contraceptive methods and IUCDs as future contraceptive options. At that time, she chose to use oral contraceptives.

## 3. Discussion

The most widely used reversible method of contraception is the intrauterine contraceptive device (IUCD), which is also the lowest risk and least expensive. It is highly effective in controlling fertility. IUCD-related complications include uterine perforation, excessive bleeding, dysmenorrhea, spontaneous expulsion, pelvic inflammatory disease (PID), and unintended pregnancy. IUCD application has a known side effect of uterine perforation with migration into a nearby organ [7]. Uterine perforation caused by IUCD migration is a serious complication; however, it occurs very rarely, with an incidence rate of 1.3 to 1.6 per 1,000 IUCD insertions [8]. Instances of IUCD migration into various locations, including the bladder wall, gut, peritoneum, and retroperitoneal region, have been documented [9, 10]. The migration of an IUCD to the bladder, as presented in this case, is a relatively uncommon complication. Previous studies have reported that the incidence of bladder perforation after IUCD placement is below 0.5% [5]. A previous report by Akhtar et al. provided information from 25 relevant research studies related to migrated intravesical IUCDs, including patient characteristics, the time interval between insertion and symptom onset, patient symptoms, and the details and outcomes of medical treatment. The duration of IUCD migration was found to vary between 6 months and 15 years. The predominant incidence was the development of a bladder calculus due to the migration of an intrauterine contraceptive device (IUCD) in 24 out of 31 patients, accounting for 78% of cases. Among the patients, the second most prevalent conditions were IUCD implanted outside the bladder (9.6% of the total, 3 out of 31 patients), and IUCD located in the bladder without a calculus (also 9.6%, 3 out of 31 patients). One patient (1/31, 3.2%) experienced ureteric obstruction. The management was highly precise and effective in every situation, achieving a success rate of 100% (32/32). In 16 instances (53%), cystoscopic retrieval, which includes cystolitholapaxy, was the most often performed procedure. Open vesicolithotomy was performed in seven cases (24%). None of these studies revealed any significant problems during or after surgery [11].

The rapid migration observed in our patient, with the IUCD translocating from the uterus to the bladder within a single year, is particularly unusual and merits close attention. Factors such as uterine contractions, inflammatory responses, and potential anatomical variations may contribute to the displacement and subsequent migration of the IUCD [10, 12].

There are two distinct forms of uterine perforation, both of which are highly susceptible to severe consequences related to the use of IUCD. Primary perforation can occur during the insertion process and is often accompanied by intense abdominal pain. Secondary perforation is a delayed occurrence, suspected to be caused by the progressive destruction of the uterine wall due to pressure [3, 10]. Uterine perforation can lead to the IUD moving outside the uterine cavity, which is a rare but serious issue. Approximately 80% of perforated IUCD are located in the peritoneal cavity. Migration into adjacent organs is an infrequent but severe consequence following perforation. Potential migration locations may include the omentum, rectosigmoid colon, peritoneum, bladder, appendix, small bowel, adnexa, and iliac vein [3]. Perforation becomes a significant issue when the IUD is implanted by an untrained practitioner, placed in an improper location, or when the patient has a weakened uterine wall near the insertion site [8, 9]. This weakening of the uterine wall often occurs as a result of multiple pregnancies, cesarean sections, or abortions [10].

Perforation of the uterus is more likely to occur during the early stages or immediately after the placement of an IUCD. Clinicians should remain vigilant for signs of acute perforation, such as difficulty during insertion, pain, or bleeding [12, 13]. In our case, the patient became pregnant 1 year after IUCD placement, indicating a possible displacement that occurred early on. This condition is supported by the ultrasound findings and plain abdominal X-ray, which revealed the presence of the T-shaped IUCD in the pelvic area (bladder). Her cystoscopy showed that the IUCD had migrated completely into the bladder, with no signs of perforation and an intact bladder mucosa. Liu reported a similar case in which the patient experienced an unexpected pregnancy after one year with an IUCD. The patient also had symptoms such as lower abdominal pain and frequent urination. Like our case, there were no risk factors such as the previous history of cesarean section and the displacement of the IUD was not detected during prenatal care, so it was assumed that the IUD had been spontaneously expelled [13]. A similar case reported by Vahdat et al. noted that no stones were found in the bladder from an IUCD that had migrated there and been present for five years. However, there was a risk factor from previous scar defects that occurred, which resulted in bladder perforation [5].

In the specific case that we encountered, we were unable to determine the exact cause of the intrauterine IUCD migration. Several factors contribute to an increased risk of uterine perforation in this case, including inexperienced individuals performing the insertion, improper positioning of the IUCD, the rigidity of the IUCD insertion instrument, and the materials used. Expanding on the factors that increase the likelihood of uterine perforation, one significant factor is the inexperienced insertion of the IUCD by individuals without proper training or expertise. Additionally, the amount of force exerted during the insertion process can also play a crucial role. The force required for IUCD insertion typically ranges from 1.5 N to 6.5 N, but it is important to note that uterine perforation can potentially occur at forces as high as 50 N [14]. This emphasizes the importance of ensuring that qualified healthcare professionals perform IUCD insertions. Improper positioning of the IUCD, either during the initial insertion or due to subsequent movement, can also increase the risk of migration beyond the uterine cavity [13]. Further research is needed to elucidate the underlying mechanisms and identify specific risk factors associated with rapid IUCD migration.

The clinical presentation of a migrated IUCD can vary. In some cases, it may be incidentally discovered during routine evaluations without any preceding symptoms. In cases where an intrauterine device (IUCD) migrates into the bladder (intravesical migration), patients may experience symptoms of urinary tract infection (UTI) that do not respond well to antibiotic treatment. These symptoms can include a sense of urgency, increased urinary frequency, hematuria (blood in the urine), or vaginal discharge. This refractoriness of UTI symptoms to antibiotics can be attributed to the presence of the migrated IUCD [6]. In our case, the patient presented with recurrent cystitis (inflammation of the bladder). The presence of copper-containing IUCD can increase the likelihood of inflammatory reactions in the surrounding tissues. The migration of such devices into the bladder can exacerbate the inflammatory response. Therefore, it is important to address this situation promptly to prevent further complications and minimize the potential for ongoing inflammation [6].

The diagnosis of a migrated IUCD may require a combination of imaging and endoscopic techniques. Imaging techniques are useful in identifying foreign bodies within the bladder [15]. For example, a full bladder ultrasound allows for the visualization of the IUCD, as demonstrated in our specific case. However, cystoscopy remains essential for a comprehensive evaluation of the bladder. It not only aids in determining the presence or absence of foreign bodies, such as bladder stones, but also assists in assessing the extent of migration within the bladder, whether partial or complete [6]. In situations where a fistula is suspected, a blue methylene test or cystography is often utilized to rule out its presence. These diagnostic tools and procedures play a crucial role in accurately diagnosing and evaluating a migrated IUCD [11, 15].

The removal of the migrated IUCD is necessary not only to alleviate symptoms and resolve the refractory UTI but also to prevent potential complications. Leaving the migrated IUCD in the bladder can lead to various problems, including persistent infections, recurrent UTIs, bladder irritation, and the formation of bladder stones [14]. Additionally, there is a risk of further migration or damage to the bladder wall, potentially leading to the development of urogenital fistulas or other serious complications. The most common method for removing a fully migrated IUCD from the bladder is through cystoscopy, a procedure that allows for direct visualization and access to the bladder [5, 6]. In most cases, similar to our specific case, we often encounter minimal difficulties during the removal process. However, in some instances where the IUCD has become calcified, lithotripsy may be performed prior to its extraction. In more complex scenarios where the IUCD has migrated into the bladder and ascended into the ureter, a cystotomy or even a laparotomy may be necessary [3].

This case serves as evidence that IUCDs can migrate to the bladder within a relatively short period. When the patient conceived after just one year, it should have prompted a thorough investigation to locate the missing IUCD. Ideally, the patient should have conducted self-checks and received a 6-week follow-up examination after the initial device insertion. Unfortunately, this case describes a suboptimal care scenario, where the patient lacked comprehensive monitoring and oversight following IUCD placement. The rapid migration of the IUCD to the bladder highlights the importance of close clinical vigilance and timely intervention. Clinicians should maintain a high index of suspicion for IUCD migration when patients present with persistent or recurrent lower urinary tract symptoms, particularly in cases where the initial insertion was performed by less experienced providers or in settings with limited access to specialized gynecological care. Prompt diagnosis and appropriate management are crucial to prevent further complications and improve patient outcomes.

## 4. Conclusions

Regular monitoring of the IUCD location helps identify any potential issues, such as migration or displacement. This proactive approach ensures early detection and prompt intervention if necessary. Performing pelvic imaging is a valuable diagnostic tool to confirm the location of the IUCD and exclude the possibility of migration to other anatomical structures. When patients with an IUCD present with lower urinary tract symptoms, it is essential to consider the potential involvement of the bladder. IUCD perforation into the bladder can lead to a range of symptoms that affect urinary function. Intravesical migration of an IUCD can result in antibiotic-refractory symptoms of UTI. Prompt removal of the migrated IUCD is essential to address the underlying cause and prevent potential complications. While such cases are relatively rare, clinicians specializing in urology and gynecology should be well-informed about this potential complication. Maintaining awareness enables timely diagnosis and appropriate management for these patients.
